# Aptamer-Functionalized Nanoparticles Mediate PD-L1 siRNA Delivery for Effective Gene Silencing in Triple-Negative Breast Cancer Cells

**DOI:** 10.3390/pharmaceutics14102225

**Published:** 2022-10-18

**Authors:** Simona Camorani, Silvia Tortorella, Lisa Agnello, Chiara Spanu, Annachiara d’Argenio, Roberto Nilo, Antonella Zannetti, Erica Locatelli, Monica Fedele, Mauro Comes Franchini, Laura Cerchia

**Affiliations:** 1 Institute of Experimental Endocrinology and Oncology “G. Salvatore” (IEOS), National Research Council (CNR), 80131 Naples, Italy; 2 Department of Industrial Chemistry Toso Montanari, University of Bologna, 40136 Bologna, Italy; 3 Department of Precision Medicine, University of Campania “L. Vanvitelli”, 80138 Naples, Italy; 4 Institute of Biostructures and Bioimaging (IBB), National Research Council (CNR), 80145 Naples, Italy

**Keywords:** aptamer, PLGA-b-PEG nanoparticles, active siRNA delivery, PD-L1, endosomal escape, TNBC

## Abstract

Small interfering RNA (siRNA) therapies require effective delivery vehicles capable of carrying the siRNA cargo into target cells. To achieve tumor-targeting, a drug delivery system would have to incorporate ligands that specifically bind to receptors expressed on cancer cells to function as portals via receptor-mediated endocytosis. Cell-targeting and internalizing aptamers are the most suitable ligands for functionalization of drug-loaded nanocarriers. Here, we designed a novel aptamer-based platform for the active delivery of siRNA targeting programmed cell death-ligand 1 (PD-L1) to triple-negative breast cancer (TNBC) cells. The generated nanovectors consist of PLGA-based polymeric nanoparticles, which were loaded with PD-L1 siRNA and conjugated on their surface with a new RNA aptamer, specific for TNBC and resistant to nucleases. In vitro results demonstrated that these aptamer-conjugated nanoparticles promote siRNA uptake specifically into TNBC MDA-MB-231 and BT-549 target cells, along with its endosomal release, without recognizing non-TNBC BT-474 breast cancer cells. Their efficiency resulted in an almost complete suppression of PD-L1 expression as early as 90 min of cell treatment. This research provides a rational strategy for optimizing siRNA delivery systems for TNBC treatments.

## 1. Introduction

Triple-negative breast cancer (TNBC), which constitutes 10% to 20% of all breast cancers, is one of the most aggressive subtypes of breast cancer with the worst outcome [1,2]. Treatment of TNBC is still challenging due to its high level of biological and clinical heterogeneity and very limited targeted treatment options due to a lack of estrogen receptor (ER), progesterone receptor (PR), and epidermal growth factor receptor 2 (HER2) and the poor availability of druggable targets [3,4]. So far, non-specific chemotherapy represents the standard of care for both early stage and advanced tumors, although its efficacy is limited by high toxicity in normal cells, poor bioavailability, and drug resistance [3,4]. Therefore, there is an unmet clinical need to identify novel agents for specific TNBC targeting and treatment.

Programmed death-1 (PD-1) is a prominent immune checkpoint receptor, which is expressed on T cells and interacts with its ligand PD-L1 on cancer cells to induce inhibitory responses, which could promote immune evasion and tumor progression [5]. PD-L1 is highly expressed by both tumor-infiltrating immune cells and tumor cells in most solid tumors, including TNBC [6,7,8], and the CD274 gene, which encodes PD-L1, is amplified in most cases of TNBC and is associated with poor outcome, highlighting a key role of the PD-L1/PD-1 axis in the suppression of anti-tumor immunity in these tumors [9,10,11]. Furthermore, exposure to chemotherapy has been shown to induce the enrichment of PD-L1-positive immune-evasive TNBC cells, which may account for the aggressive behavior of recurrent and metastatic tumors that survive chemotherapy [12]. Accordingly, the humanized IgG1 monoclonal antibody (mAb) Atezolizumab, targeting PD-L1, has entered the clinic as a viable treatment, in combination with nab-paclitaxel, for locally advanced or metastatic TNBC [13]. Nevertheless, serious drawbacks still remain for mAb-based treatment, mainly due to the inaccessibility of cytoplasmic PD-L1, which has been shown to play intrinsic pro-tumorigenic roles [14,15], and the limited efficiency in cases with low PD-L1 expression [16]. Those and other issues, including the time- and cost-consuming production of mAbs, their potential for immunogenicity, and low shelf-life, can be overcome by suppression of PD-L1 through gene silencing. Selective gene silencing through RNA interferences has been widely and successfully employed in functional studies and is currently being investigated as a potential tool for cancer treatment [17]. Small interfering RNA (siRNA), short hairpin RNA, and their optimized chemical modifications are the active silencing agents and are effective in targeting individual mRNAs in a specific way. However, due to the highly charged nucleic acid backbone, the development of effective and safe tools for the selective delivery of these molecules into tumor cells is recognized as a key step towards their eventual development as therapeutics. The improvement of the transport of the above therapeutics by safe biodegradable nanocarriers will therefore enhance the efficacy of the treatment [18]. Advances in nanocarriers’ formulations have recently enabled several clinical trials in which siRNAs have been systemically administered to cancer patients [18].

Poly(lactic-co-glycolic)-block-poly ethylene glycol (PLGA-b-PEG) has gained great attention in recent decades as a major component for nanovectors, because of its capability to create biodegradable polymeric nanoparticles (PNPs), which are congenial to targeted drug delivery approaches: indeed, thanks to its amphiphilic property, PLGA-b-PEG is able to self-assemble under mild conditions into a micellar-like structure, protecting the drug, siRNA, or other small molecules in its core while exposing the PEG shell to the external environment, thus ensuring the dispersibility of the whole nanosystem in water or the physiological environment [19,20]. Moreover, both PEG and PLGA are known to be non-toxic, generally recognized as safe (GRAS), and Food and Drug Administration (FDA)-approved for medical purposes [19,20].

These nanosystems have been long used for the trapping of many anti-cancer drugs (such as Dactolisib [21] or Cisplatin [22]) and also siRNA molecules by means of different preparation methods such as nanoprecipitation, solvent displacement, or single-/double-emulsions [23,24,25].

Indeed, PLGA-b-PEG offers the possibility to tune the affinity of its core towards hydrophilic or hydrophobic molecules simply by modifying the micelle preparation procedure: for instance, by applying a nanoprecipitation or oil-in-water emulsification protocol, PLGA remains confined in the core, thus hosting hydrophobic moieties; on the contrary, by applying a water-in-oil-in-water double-emulsion protocol, the copolymers chains create a bilayer structure, similar to that of cellular membranes, whereas PEG is oriented both towards the inner and the external side, with PLGA chains forming a middle bilayer shell [26,27]. The latter conformation is suitable for hosting, protecting, and delivering hydrophilic moieties, such as siRNA, which should neither be dispersed into the blood stream, nor administered systemically [26,27].

Here, we report a new aptamer-targeted nanosystem for efficient delivery of siRNA molecules designed to suppress PD-L1 expression (siPD-L1) specifically into TNBC cells. The sTN145 2’Fluoro-pyrimidines (2’F-Py) RNA aptamer, which we generated from TNBC cell-SELEX [28,29], was used as a TNBC-targeting ligand to confer tumor selectivity to nanoparticles loaded with siPD-L1. The aptamer binds to a not-yet-known protein expressed on the surface of TNBC cells and actively internalizes into target cells [28,29], thus representing an optimal tool for enabling active targeting of TNBC. Importantly, sTN145 exhibits unequivocal efficacy in targeting human cell lines and tissues covering different TNBC subtypes by discriminating them from both normal samples and triple-positive breast cancers (TPBC: ER+, PR+, HER2 over-expression). In addition, according to the 2’F-Py modification, it shows superb nuclease resistance [29].

In this study, we developed novel PNPs decorated with the sTN145 aptamer and loaded with siPD-L1, succeeding in highly efficient siRNA entrapment. We show that, according to the TNBC cell type specificity of the sTN145 aptamer, the aptamer-decorated PNPs efficiently deliver the siRNA cargo into TNBC MDA-MB-231 and BT-549 cells, but not into TPBC cells. Importantly, the siRNAs delivered by the sTN145-aptamer-decorated nanoparticles resulted in efficient PD-L1 gene silencing and suppression of PD-L1-induced epithelial–mesenchymal transition (EMT) factor Snail in TNBC cells.

Taken together, these results present a viable platform for the active delivery of siRNA to cancer cells and strongly support further investigation to evaluate its efficacy for boosting anti-tumor immunotherapy in TNBC.

## 2. Materials and Methods

### 2.1. Aptamers and siRNAs

NH_2_-terminated 2’F-Py-containing RNA sTN145 and scrambled (SCR) aptamer used as a control were synthesized by LGC Biosearch Technologies (Risskov, Denmark). The sequences of the aptamers were previously reported [29].

Human PD-L1 siRNA sequences entrapped in nanoparticles (Hs-CD274_1, referred to as siPD-L1) were: sense sequence 5’-GUAGCAAUAUGACAAUUGATT-3’ and anti-sense sequence 5’-UCAAUUGUCAUAUUGCUACCA-3’. siPD-L1 and its 5’ Fluorescein (6-FAM)-labeled version (FAM-siPD-L1) were purchased from Qiagen (Hilden, Germany).

### 2.2. Synthesis of Nanoparticles

The preparation of the PLGA-PEG nanoparticles loaded with siPD-L1 followed a previously reported procedure [21,22]. Briefly, 100 mg of PLGA-b-PEG-COOH was dissolved in 10 mL of chloroform and mixed with 1 mL of a solution of siPD-L1 and Poly-D-Lysine (PDL) (molar ratio *w/w* 6:1). Then, the two-phase mixture was emulsified, in an ice bath, with a tip probe sonicator (600 W input, 40% ampl) for 45 s. Subsequently, 144 mL of 1.25% sodium cholate solution in water was slowly added to the obtained emulsion: the resulting two-phase mixture was further emulsified for 3 min, in an ice bath, at the amplitude shown above. The FAM-siPD-L1@PNPs were likewise prepared.

After the final water-in-oil-in-water (w1/o/w2) emulsion thus created was ready, the chloroform was completely evaporated under reduced pressure, and the resulting particles were washed and concentrated using centrifugal filter devices (Amicon Ultra, Ultracell membrane with 100,000 NMWL, Millipore, Billerica, MA) to a final volume of 5 mL and, finally, filtered using nylon syringe filters (13 mm, 0.22 μm, Nazionale, Italy).

For the conjugation onto the outer shell of the aptamer sequence, siPD-L1@PNPs or FAM-siPD-L1@PNPs were diluted in H_2_O to reach a volume of 8 mL, then a solution of N-hydroxysulfosuccinimide 2.3 mM (4.3 mL) and a solution of 1-ethyl-3-(3-(dimethylamino)propyl) carbodiimide (EDC) 0.28 M (1.8 mL) were added to the vial. The -COOH activation was carried out at room temperature (RT) for 10 min, then 102 pmol of the sTN145 aptamer or of the scrambled sequence, dissolved in 1 mL of water and suitably activated with a cycle of 5′ at 85 °C, 3′ at 0 °C, and 10′ at 37 °C, was added and left to react for 24 h. After that, the particles obtained were washed and purified by repeating the same passages described above (centrifugation with filtering devices and filtration on syringe filters). The final volume was adjusted to 5 mL with sterile water.

Dynamic light scattering (DLS) analysis and ζ-potential values were acquired with a Zetasizer Nano-S (Malvern) instrument, working with a 532 nm laser beam at 25 °C, using standard cuvettes or DTS1070 Clear Disposable zeta cells, and the results are expressed as the average of three measurements.

### 2.3. Quantitative Determination of siPD-L1 Entrapped in the Polymeric Nanoparticles

The siPD-L1 encapsulation efficiency was determined by measuring the amount of extractable siRNA in the PNPs. To this aim, 0.5 mL of siPD-L1@PNPs (2 mg of dry matter) was added to 0.2 mL of chloroform and 0.25 mL of TE buffer, then rotated end-over-end for 90 min at RT to facilitate the extraction of siRNA from the organic phase in the aqueous phase. The aqueous and organic phases were separated by centrifugation for 15 min at 13,200 rpm at 4 °C. The upper aqueous phase containing the RNA was transferred to a new tube and incubated for 5 min at 37 °C to remove the residual chloroform, concentrated using centrifugal filter devices (Amicon Ultra-0.5 mL 3000 MW-cutoff centrifugal filter, Millipore), and 8 µL of each sample was loaded onto the denaturing PAGE (15% polyacrylamide with 7 M urea). The gel was stained with ethidium bromide and UV exposed to visualize the RNA bands. Band intensity was quantified using ImageJ (v1.46r), and the amount of siPD-L1 extracted was extrapolated from a linear standard curve obtained with different amounts of siPD-L1.

Another determination, albeit indirect, of the amount of FAM-siPD-L1 was assessed by quantitative fluorescent analysis using an Edinburgh FLSP920 spectrofluorimeter equipped with a 450 W Xenon arc lamp. The measurement was performed using a subtractive strategy, checking the fluorescence emission of the PNPs’ washing waters after each fabrication/conjugation step. The spectrofluorimetric determination was performed using an excitation wavelength of 485 nm and an emission wavelength of 520 nm and FAM-siPD-L1 standard solutions at different concentrations (1 to 20 nM) in water.

### 2.4. Cell Cultures

Human BT-549 and MDA-MB-231 (TNBC) and BT-474 (TPBC) cell lines (American Type Culture Collection, Manassas, VA) were grown as previously reported [29,30].

### 2.5. Cell Transfection

BT-549 and MDA-MB-231 cells (1.8 × 10^5^ cells/well, 6-well plates) were transfected for 5 h at 37° C with PD-L1 siRNA or control siRNA (siRNA ctrl), purchased from Qiagen (Hilden, Germany), by using Lipofectamine RNAiMAX Reagent (Life technologies, Milan, Italy), according to the manufacturer’s instructions. At 48 h post-transfection, cell lysates were prepared and analyzed by immunoblot.

### 2.6. Immunoblot

Cell lysates’ preparation and immunoblot analyses were performed as previously reported [31]. The filters were probed with the indicated primary antibodies: anti-PD-L1 (E1L3N), anti-Snail (Cell Signaling Technology Inc., Danvers, MA), anti-vinculin (Santa Cruz Biotechnology, Santa Cruz, CA), and anti-GAPDH (Sigma-Aldrich, Milan, Italy). Densitometric analysis was performed on at least two different exposures to ensure the linearity of each acquisition using ImageJ (v1.46r). The blots shown are representative of at least three independent experiments.

### 2.7. Cell Viability Assays

MDA-MB-231 and BT-549 cells (5.0 × 10^3^ cells/well, 96-well plates) were treated with empty@PNPs, empty@PNPs-SCR, or empty@PNPs-sTN145 (0.5 and 1 mg/mL dry matter) for 24 h, and cell viability was assessed by Thiazolyl Blue Tetrazolium Bromide (MTT, AppliChem GmbH, Darmstadt, Germany), according to the manufacturer’s protocol.

### 2.8. Confocal Microscopy

Cell internalization experiments were performed as previously reported [32]. Briefly, TNBC BT-549 cells (8.0 × 10^4^ cells/well) were seeded on glass coverslips placed in a 24-well plate one day prior to the experiment. Subsequently, the medium was removed and the cells were stained live with LysoTracker Red DND-99 (1:1000, Invitrogen, Carlsbad, CA, USA) in Roswell Park Memorial Institute-1640 medium (RPMI-1640, Sigma-Aldrich) supplemented with 10% fetal bovine serum (Sigma-Aldrich) for 1 h at 37 °C. After three washes with Dulbecco’s phosphate-buffered saline solution (DPBS, Sigma-Aldrich), the cells were incubated for 0.5, 1, 2, and 5 h at 37 °C with FAM-siPD-L1@PNPs, FAM-siPD-L1@PNPs-SCR, or FAM-siPD-L1@PNPs-sTN145 (60 nM final siRNA concentration) diluted in RPMI-1640 supplemented with 0.1 mg/mL yeast tRNA and 0.1 mg/mL ultrapure™ salmon sperm DNA (Invitrogen), as nonspecific competitors. After washing, cells were fixed with 4% paraformaldehyde in DPBS for 20 min and incubated with 1.5 µM 4’,6-Diamidino-2-phenylindole (DAPI, D9542, Sigma-Aldrich) in DPBS for 5 min, and coverslips were mounted with glycerol/DPBS.

TPBC BT-474 cells (8.0 × 10^4^ cells/well, 24-well plate) incubated for 1 h at 37 °C with FAM-siPD-L1@PNPs, FAM-siPD-L1@PNPs-SCR, or FAM-siPD-L1@PNPs-sTN145 as above served as the nontarget cells.

The samples were visualized using Zeiss LSM 700 META confocal microscopy equipped with a Plan-Apochromat 63×/1.4 Oil DIC objective. Manders’ colocalization coefficients M1 and M2 were calculated by the Zeiss Software.

### 2.9. Statistical Analysis

Statistical values were defined using GraphPad Prism version 6.00 by one-way ANOVA followed by Tukey’s multiple comparison test. A *p*-value < 0.05 was considered significant for all analyses.

## 3. Results

### 3.1. Synthesis of Aptamer-Decorated and siRNA-Loaded Nanosystems

First, we tested four validated PD-L1 siRNAs (30 nM final concentration) for the silencing power of the PD-L1 gene by cell transfection experiments in both TNBC MDA-MB-231 and BT-549 cells. These cell lines, which express a high level of PD-L1 (Figure S1 and [7,31,33]), have been used as a model system because they are efficiently targeted by the sTN145 aptamer, which is rapidly internalized into both cell lines [28,29]. Among the siRNAs tested, we selected Hs_CD274_1 (herein referred to as siPD-L1) to be entrapped into PNPs, which provided a reduction of at least 70% in the PD-L1 protein levels, as assessed by immunoblot at 48 h post-transfection (Figure S1A). A fluorescein-labeled version of siPD-L1 (FAM-siPD-L1), which retains the efficiency of unlabeled siRNA (Figure S1B), was used for visualization purposes by confocal microscopy.

**Figure 1:**
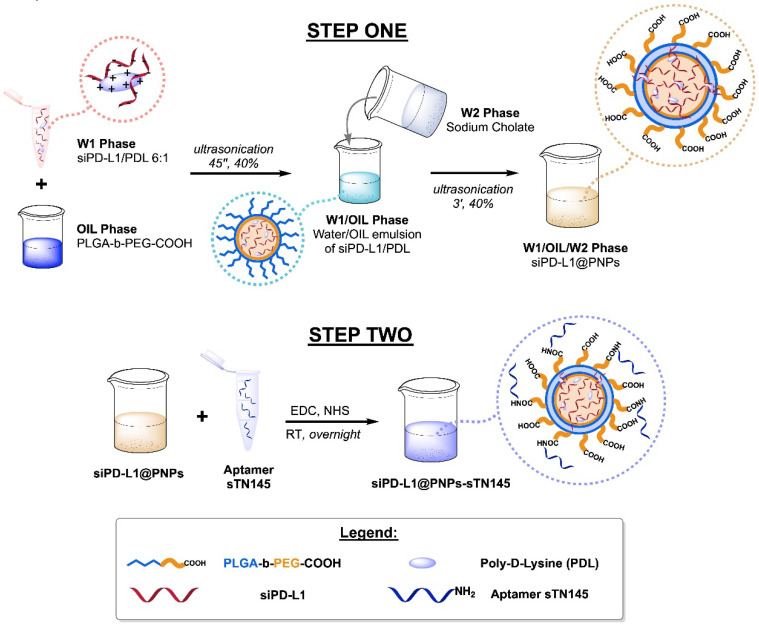
Schematic representation of the water-in-oil-in-water workflow for obtaining the final PNPs. Encapsulated siRNA and covalently labeled aptamer molecules (and scrambled sequence) were used for loading/decorating multifunctional nanovectors.

PNPs systems were prepared according to the protocol already described [21,22] with some modifications to allow for more efficient siRNA entrapment (Figure 1). PNP fabrication begins with the synthesis of the PLGA-b-PEG-COOH copolymer, following a previously reported procedure [19]. After obtainment of the copolymer, the water-in-oil-in-water (w1/o/w2) double-emulsion sonication method [34] was chosen as the strategy for the particles’ fabrication; thanks to this protocol, we entrapped siPD-L1 (or FAM-siPD-L1) in the hydrophilic core of PNPs, obtaining a final formulation dispersible in water. Since the overall charge of siPD-L1 is negative, a cationic modification using PDL was introduced prior to the emulsification procedure. PDL was employed to complex siPD-L1 and decrease repulsion towards the hydrophobic PLGA matrix; thus, the resulting PDL/siPD-L1 complexes were more efficiently loaded into the PLGA-b-PEG-COOH nanoparticles. After the purification of the obtained siPD-L1@PNPs, EDC chemistry was adopted to carry out the conjugation of amino-terminated sTN145, exploiting the residual carboxylic groups onto the outer layer of the PNPs, derived from the PEG chains. The nanosystem thus created was named siPD-L1@PNPs-sTN145. In order to demonstrate that there is a true correspondence between the results obtained and the presence of a TNBC-specific aptamer onto the nanoparticle shell, the labeling was also performed with a scrambled sequence (SCR) used as a negative control (referred to as siPD-L1@PNPs-SCR).

### 3.2. Characterization of Aptamer-Decorated and siRNA-Loaded Nanosystems

DLS revealed FAM-siPD-L1@PNPs-sTN145 and siPD-L1@PNPs-sTN145 diameters, respectively equal to 113.1 ± 0.96 and 98.08 ± 0.49 nm, a low polydispersity index (PDI = 0.172 and 0.159), and a negative ζ-potential value of −16.7 mV and −23.6 mV, due to unreacted carboxylic acid groups, which derive from PEG chains and remain free even after the conjugation protocol, ensuring thus a slightly negative surface charge, which guarantees an optimal and efficient dispersion of the entire nanosystem in water and physiological solution. On the other hand, the particle size of the SCR-labeled system was 100.1 ± 0.67 nm (FAM-siPD-L1 loaded) and 98.43 ± 1.88 nm (siPD-L1 loaded), with a PDI average equal to 0.165 and a negative ζ-potential value around −20 mV. In order to confirm whether all nanosystems remain stable and do not undergo any physicochemical alterations, a certain amount of PNP solutions was collected, suitably diluted, and stored at 4 °C for four weeks. No significant variation was noted in the three values of the above parameters (DLS, PDI, and ζ-potential). Furthermore, the macro morphological aspect of the colloidal solution was still clear, opalescent, free of precipitates, and homogenous. The final overall concentration of siPD-L1@PNPs-sTN145 or SCR in solution was determined by gravimetric analysis by drying 100 µL of solution at 120 °C for 24 h and then carefully weighing the amount of residual dry matter. The results showed a final concentration of 4 to 10 mg/mL for all samples; the amount of siPD-L1 (or FAM-siPD-L1) entrapped in the PNPs was calculated (see Materials and Methods and Figure S2 for details). The characterization results obtained for all the synthesized nanosystems are summarized in Table 1.

**Figure 2:**
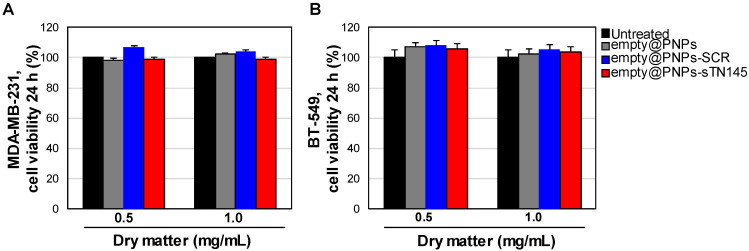
Aptamer-decorated PNPs are not cytotoxic. MDA-MB-231 (**A**) and BT-549 (**B**) cells were left untreated or treated for 24 h with the indicated amount of unloaded PNPs (empty@PNPs, empty@PNPs-SCR or empty@PNPs-sTN145). Cell viability was determined and expressed as the percent of viable treated cells with respect to untreated cells. Bars depict means ± SD of three independent experiments.

In vitro basal cytotoxicity tests in MDA-MB-231 and BT-549 cells, specifically targeted by the sTN145 aptamer [28,29], revealed the safety profile of the unloaded PNPs, unconjugated and conjugated with either sTN145 or SCR aptamers, up to 1 mg/mL (Figure 2), the maximum carrier concentration used in the functional studies.

### 3.3. Selective Uptake of siPD-L1@PNPs-TN145 in TNBC Cells

**Figure 3:**
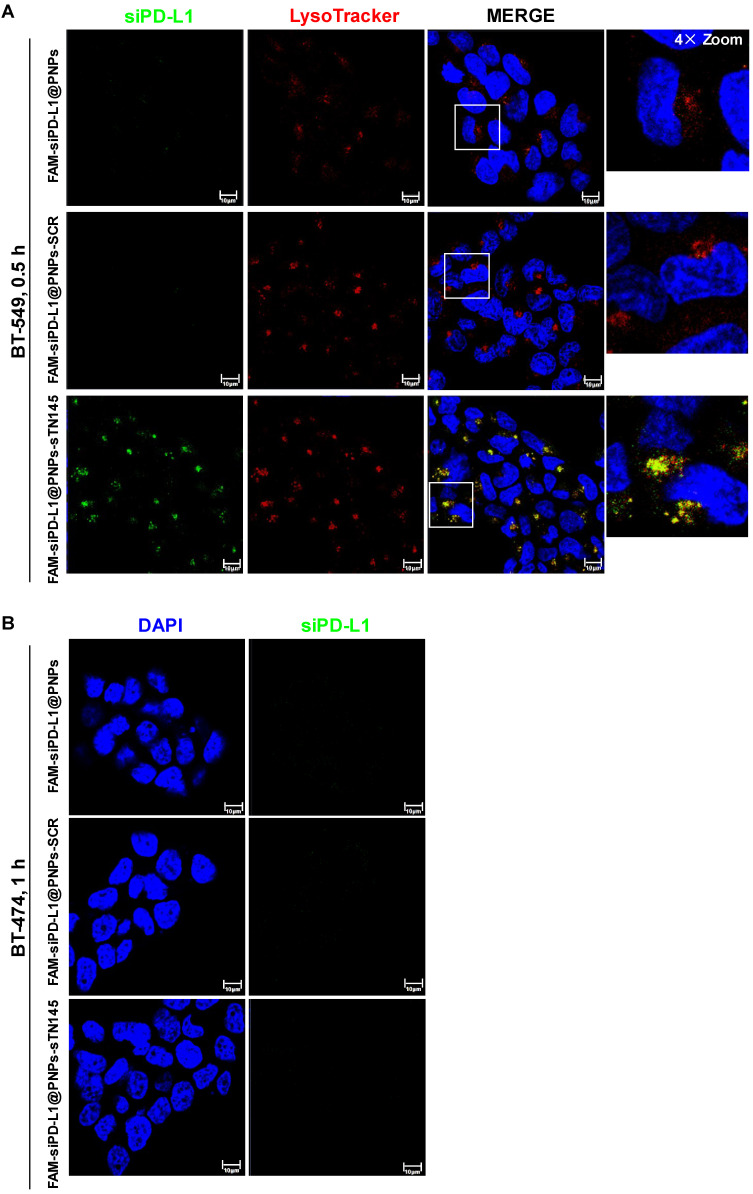
FAM-siPD-L1@PNPs-sTN145 are effectively taken up by TNBC BT-549 cells. (**A**) Representative confocal images of BT-549 cells incubated for 0.5 h at 37 °C with FAM-siPD-L1@PNPs, FAM-siPD-L1@PNPs-SCR, or FAM-siPD-L1@PNPs-sTN145 after live-staining with LysoTracker Red for endosomes’ visualization. FAM-siPD-L1, LysoTracker, and nuclei are visualized in green, red, and blue, respectively. Co-localization results appear yellow in the merged images. White squares indicate the area shown in insets in a magnified view obtained using Image J software. (**B**) Representative confocal images of TPBC BT-474 cells incubated for 1 h at 37 °C with FAM-siPD-L1@PNPs, FAM-siPD-L1@PNPs-SCR, or FAM-siPD-L1@PNPs-sTN145. FAM-siPD-L1 and nuclei are visualized in green and blue, respectively. (**A**,**B**) Magnification 63×, 1.0× digital zoom, scale bar = 10 μm. Inset: 4× digital zoom. All digital images were captured at the same setting to allow direct comparison of staining patterns.

**Figure 4:**
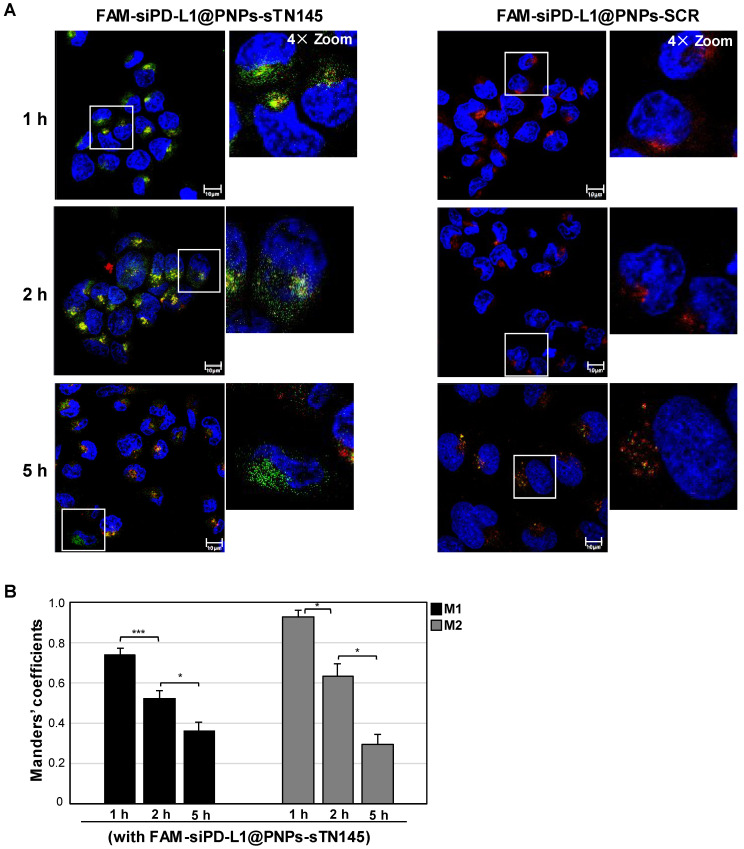
Selective cell uptake and release from endosomes of FAM-siPD-L1@PNPs-sTN145 compared to FAM-siPD-L1@PNPs-SCR. (**A**) Representative merged confocal images of BT-549 cells incubated for different periods (1 to 5 h) at 37 °C with FAM-siPD-L1@PNPs-sTN145 or FAM-siPD-L1@PNPs-SCR after live-staining with LysoTracker Red for endosomes’ visualization. FAM-siPD-L1, LysoTracker, and nuclei are visualized in green, red, and blue, respectively. Co-localization results appear in yellow in the images. The white squares indicate the area shown in insets in a magnified view obtained using Image J software. Magnification 63×, 1.0× digital zoom, scale bar = 10 μm. Inset: 4× digital zoom. All digital images were acquired with the same setup to allow direct comparison of staining patterns. (**B**) Quantitative analysis of colocalization of FAM-siPD-L1, in the presence of FAM-siPD-L1@PNPs-sTN145 treatment, with endosomes. Manders’ coefficient M1 quantifies the fraction of FAM-siPD-L1 signal overlapping with endosomal signal; M2 quantifies the fraction of endosomal signal overlapping with FAM-siPD-L1. M1 and M2 were quantified on 10 separate images for each incubation time. Bars depict means ± SD. * *p* < 0.05, *** *p* < 0.001 on a minimum of 100 cells for each condition.

Nanocarriers, left unconjugated and conjugated to sTN145 or SCR, were first monitored for targeting specificity and internalization efficiency into target cells using FAM-siPD-L1 as a cargo. Confocal microscopy analyses revealed that the sTN145 aptamer is able to specifically target siRNA-loaded nanovectors to BT-549 cells and promote their rapid cell uptake, as demonstrated by the clear detection of the siRNA-associated signal (green in the confocal microscopic images) in the cells after 0.5 h of incubation with the targeted FAM-siPD-L1@PNPs-sTN145, but not the non-targeted ones, both unconjugated (FAM-siPD-L1@PNPs) and decorated with the scrambled aptamer (FAM-siPD-L1@PNPs-SCR) (Figure 3A). Furthermore, at this time point, the fluorescent signal from the FAM dye co-localized in sharp yellow spots with LysoTracker, which stains the acidic cellular organelles (red in the confocal microscopic images), indicating that the FAM-siPD-L1@PNPs-sTN145 were entrapped in the endosomes (Figure 3A). In agreement with these observations, the Manders’ coefficients (M1 and M2), used to quantify the degree of co-localization, were 0.86 and 0.56, respectively, thus confirming that most of the detected nanoparticles were present in the endosomes and some of these organelles did not include nanoparticles. By evaluating the fate of FAM-siRNA at increasing incubation times of the nanoparticles up to 5 h, the FAM signal became progressively more delocalized from LysoTracker signal, suggesting the successful escape of the FAM-siRNA from endosomes into the cytoplasm (Figure 4A,B and Figure S3).

Conversely, siPD-L1-associated signals were undetectable with SCR-decorated nanovectors for up to 1 h and slightly visible from 2 h incubation (Figure 3A, Figure 4A and Figure S3). According to the targeting specificity of the sTN145 aptamer for TNBC cells [28,29], no fluorescent signal from FAM-siPD-L1@PNPs-sTN145 was observed with TPBC BT-474 cells, used as a negative control (Figure 3B).

Overall, these results indicate that the nanoparticles’ cell uptake is driven by the sTN145 aptamer, which retains its binding-competent folding after conjugation onto the outer surface of siRNA-loaded PNPs.

### 3.4. Efficient PD-L1 Gene Silencing by siPD-L1@PNPs-TN145

**Figure 5:**
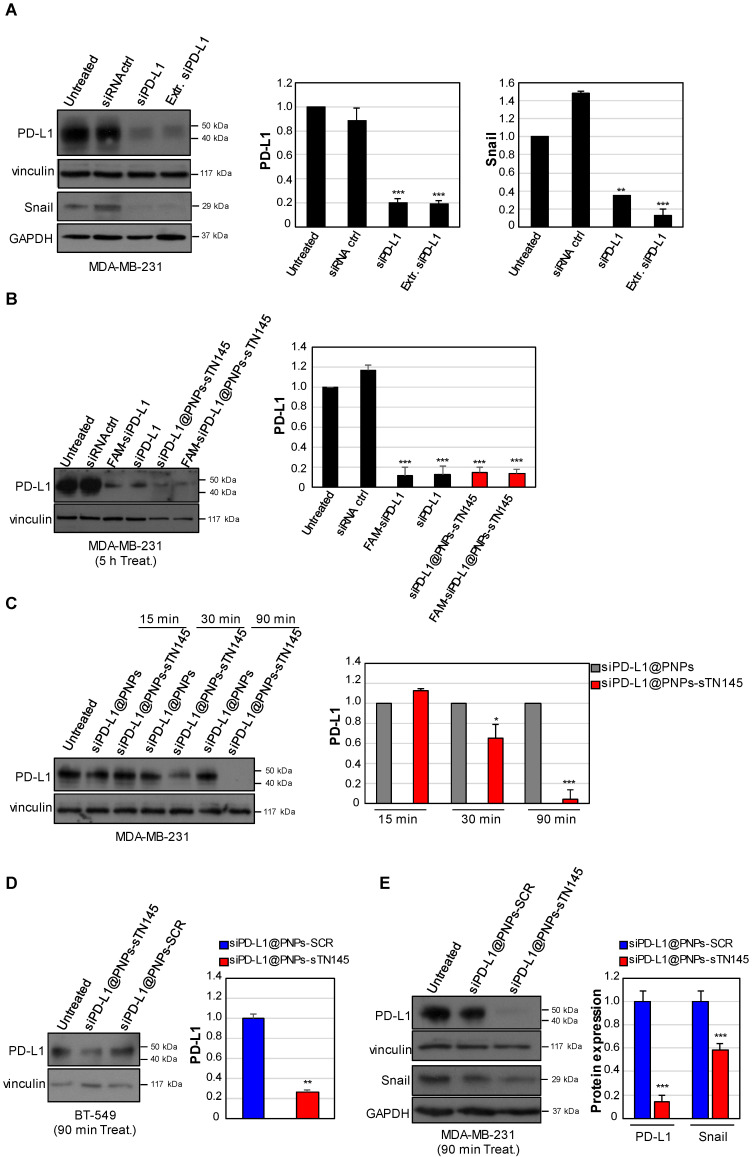
sTN145-conjugated nanoparticles deliver PD-L1 siRNA into TNBC cells. MDA-MB-231 cells were left untreated or transfected with 30 nM PD-L1 siRNA either naked (siPD-L1), FAM-labeled (FAM-siPD-L1), or extracted from siPD-L1@PNPs (Extr. siPD-L1), using Lipofectamine reagent. Nonsilencing siRNA (siRNA ctrl) was used as a control (**A**,**B**). MDA-MB-231 (**B**,**C**,**E**) and BT-549 (**D**) cells were left untreated or treated with the indicated nanovectors (30 nM siRNA final concentration) for the indicated times. (**A**–**E**) After washes and 48 h of recovery, cells were harvested and cell lysates prepared and immunoblotted with anti-PD-L1 and anti-Snail antibodies, as indicated. Anti-vinculin or anti-GAPDH antibodies were used as a control for immunoblot loading. The molecular weights of the indicated proteins are reported. The histograms indicate the PD-L1/vinculin (**A**–**E**) and Snail/GAPDH (**A**,**E**) ratio of the densitometric signals. Values are shown relative to untreated (**A**,**B**) or treatment with untargeted nanovectors, siPD-L1@PNPs (**C**) and siPD-L1@PNPs-SCR (**D**,**E**), arbitrarily set to 1. Bars depict means ± SD of three independent experiments. * *p* < 0.05, ** *p* < 0.01, *** *p* < 0.001.

As a next step, we asked whether the siPD-L1 cargo delivered into TNBC cells by the sTN145-decorated nanoparticles reduces the level of PD-L1. To this aim, we first assessed that the functional activity of siPD-L1 entrapped in the nanoparticles was not affected by the nanovector preparation procedure. Therefore, siRNA molecules were extracted by siPD-L1@PNPs and transfected into MDA-MB-231 cells (30 nM siRNA concentration) via the Lipofectamine RNAiMax transfection reagent, and the PD-L1 protein levels were determined after 48 h by immunoblot. As shown (Figure 5A), the extracted siPD-L1 caused PD-L1 silencing comparable to that achieved by the siPD-L1 positive control used under the same experimental conditions, thus indicating that it retains in the nanovector a state competent to cause effective PD-L1 silencing. Furthermore, in agreement with the established tumor-intrinsic role of PD-L1 in promoting EMT in TNBC cells [33], which is independent of its role as an immune checkpoint, both the siPD-L1 positive control and the extracted siPD-L1 strongly reduced the protein levels of the EMT transcription factor Snail (Figure 5A).

Next, in order to compare the silencing effect of siPD-L1 delivered by the sTN145-equipped PNPs with that of the same amount of transfected siRNA, MDA-MB-231 cells were treated for 5 h with siPD-L1@PNPs-sTN145 (30 nM final siRNA concentration) or transfected with siPD-L1, washed, and left untreated for a further 48 h. As shown in Figure 5B, the siPD-L1@PNPs-sTN145 treatment resulted in an efficient reduction of the PD-L1 protein, to levels comparable to those found upon transfection with the siPD-L1 moiety (about 80%), compared to cells not treated or treated with the control nonsilencing siRNA. As expected, similar results were obtained in the presence of the FAM-siPD-L1@PNPs-sTN145 treatment. Thus, to gain deeper insight into the timing of the active delivery of siPD-L1 by the sTN145-decorated and siRNA-loaded nanosystems, MDA-MB-231 cells were treated with either siPD-L1@PNPs-sTN145 or unconjugated siPD-L1@PNPs (30 nM final siRNA concentration) for 15, 30, and 90 min, washed, and left untreated for a further 48 h. As shown in Figure 5C, the PD-L1 protein levels were reduced by approximately 30% starting with 30 min of cell treatment with siPD-L1@PNPs-sTN145, compared to the non-targeted control nanovectors, and nearly undetectable as early as at 90 min of treatment. These results were confirmed in BT-549, where an approximately 70% reduction in the PD-L1 protein levels was observed after 90 min of treatment with siPD-L1@PNPs-sTN145, while no effect was elicited by siPD-L1@PNPs-SCR (Figure 5D), according to the confocal microscopy analyses, which showed the selective uptake of nanovectors driven by the sTN145, but not by the scrambled aptamer. Furthermore, siPD-L1@PNPs-sTN145, unlike siPD-L1@PNPs-SCR, efficiently reduced the expression of the PD-L1 downstream effector Snail (Figure 5E).

These results indicate that sTN145-conjugated nanoparticles effectively deliver a functional PD-L1 siRNA into TNBC target cells.

## 4. Discussion

Using cell-SELEX, we recently generated and characterized an aptamer, termed sTN145, which targets a membrane protein uniquely expressed on the surface of TNBC cells with high affinity and selectivity [28,29]. Here, this aptamer has been attached to the surface of polymeric nanoparticles, entrapping siRNA targeted at PD-L1, to promote their uptake and delivery of their cargo into TNBC target cells.

Aptamers targeting cancer cell surface proteins have emerged as a viable alternative or complement to conventional antibodies for active cancer targeting due to their small size, which improves tissue penetration and their easy production through chemical synthesis [35]. Aptamers support a variety of chemical modifications to increase their half-life, reduce toxicity, and allow for combination therapy by conjugating them with drugs or different drug-loaded nanoformulations. Most importantly, aptamers are safe biomolecules with no immunogenicity [36]. Aptamer-escorted drug-loaded nanoparticles represent a promising approach for the targeted delivery of therapeutics to tumors, while sparing healthy cells, and have numerous advantages over chimeric constructs in which the drug is directly conjugated to the aptamer used for cell targeting/internalization. First, different aptamers can be attached to the surface of the nanoparticles, increasing the targeting specificity. Furthermore, the coordination of multiple therapeutic agents in a single platform can not only improve their stability, bioavailability, and pharmacokinetic profile, but also allow for the combined delivery on the desired target [37].

Various delivery systems, differing in composition, size, and chemical properties, have been developed so far, in an attempt to overcome major barriers to the use of siRNA therapies. Nevertheless, tumor-targeted delivery systems [18,38,39] still suffer from both the paucity of validated ligands that specifically bind certain tumor cell markers and the low yield of siRNA that effectively reaches its target in the cytoplasm [40,41].

Among the different nanocarriers, polymeric nanoparticles have gained increasing and constant attention in recent decades due to their effectiveness in cargo delivery, stability in different physiological media, and reliability of their behavior, release profile, and above all, safety. In particular, PLGA is probably the most-studied polymer as it has complete biodegradability, is FDA approved for medical application, and exhibits an extraordinary ability to assemble into nanometer-sized micelles, capable of trapping small molecules, such as drugs, and releasing them into the body in a time-dependent manner. Despite the excellent qualities, the use of PLGA polymeric nanoparticles for drug delivery applications still remains problematic due to the fast removal of these nanocarriers from the bloodstream by the liver and spleen, thus drastically reducing drug availability to the tumor tissue. To overcome these issues, PEG was employed, because, thanks to its “stealth behavior”, which inhibits the rapid recognition of a foreign agent by the immune system (opsonization), it is able to significantly increase the blood circulation time of the nanocarrier. Moreover, PEG is hydrophilic and able to stabilize nanoparticles through steric effects, especially in water. The PLGA-PEG block copolymer is thus an excellent system, and in the last decade, it has emerged as one of the most-promising systems for nanoparticle formation, drug loading, and drug delivery applications in vivo [19]. Furthermore, new protocols and techniques have recently been developed with the aim of making possible the entrapment into PLGA-PEG nanocarriers, not only of lipophilic molecules, but also of hydrophilic moieties [42]. This was a breakthrough for nanomedicine, as most chemotherapy drugs used in the past are lipophilic, but many recently developed agents are hydrophilic. This is the case with siRNA molecules, the application of which represents a powerful therapeutic tool against a plethora of previously untreatable diseases. One of these methodologies is the so-called water-in-oil-in-water double-emulsion, which allows for the formation of bilayer micelles, which expose the PEG both on the outer surface and in the inner core, while the PLGA remains in the middle, forming the bilayer, thus enabling the stability, in water, of water-soluble moieties, such as siRNA. siRNA entrapment into PLGA nanocarriers has been attempted and implemented in recent years [23], but none of the reported vectors have been targeted with aptamers for specific targeting of cancer or other diseases. The siRNA-loaded PLGA-b-PEG nanoparticles conjugated to the sTN145 aptamer presented here may therefore represent effective real enhancement of an already powerful weapon, but they also present a great challenge, since there is the possibility to lose siRNA from the particles during the conjugation protocol or to alter the efficacy of the siRNA or aptamer, or both agents, due to an unexpected side reaction or biological effects.

In vitro characterization of multicomponent nanovectors clearly demonstrates that the targeting agent (i.e., the sTN145 aptamer attached to the PNPs) and therapeutic cargo (i.e., the PD-L1 siRNA entrapped in the PNPs) retain their functional activity when integrated into the nanoparticle bioconjugates. This leads to the selective release of siPD-L1 in TNBC cells, due to the high binding sensitivity and specificity of sTN145 [28,29] and the consequent suppression of PD-L1, and Snail effector, expression in targeted cells. Interestingly, it has been reported that polymeric PLGA nanoparticles may rapidly escape the endo-lysosomal compartments by reversing their surface charge (from anionic to cationic) in the acidic pH of endo-lysosomes, which allows the interaction of nanoparticles with the vesicular membrane and escape into the cytosol [43,44].

Our results strongly encourage further in vivo studies with animal models to confirm the therapeutic utility of our nanovectors. Due to the capability of sTN145 to recognize its target on murine 4T1 TNBC cells (our personal communication), studies in orthotopic 4T1 xenografts in syngeneic mice are ongoing in our laboratories to evaluate the role of the immune system in the response to targeted delivery of therapeutic nanoparticles to tumor. Moreover, the identification of the surface protein that is enriched on mesenchymal TNBC MDA-MB-231 and BT-549 cells and is specifically recognized by the sTN145 aptamer will allow researchers and clinicians to apply our unique drug-loaded nanoparticles to humanized mice obtained from patient-derived TNBC xenografts and to use them as a future valuable tool for personalized treatments.

After an initial response to cytotoxic chemotherapy, a large proportion of TNBC patients often develop recurrent tumors, which are chemoresistant and highly metastatic [1,30,45]. To form a secondary (recurrent and/or metastatic) tumor, a breast cancer cell must evade the innate and adaptive immune systems. As one of the possible mechanisms explaining the detrimental effect of chemotherapeutic treatment, chemotherapy, including cisplatin administration, has been shown to induce the enrichment of evasive PD-L1+ immune TNBC cells [12]. Thus, an aptamer-targeted nanosystem that enables the synergistic effect of siRNA, which directly knocks down the expression of PD-L1 on tumor cells, with a potent chemotherapeutic drug might be a viable way to eradicate TNBC cells. In this regard, we recently used the anti-EGFR CL4 aptamer to decorate cisplatin-loaded PNPs and proved the tumor-targeting, safety profile, and anti-tumor activity of the resulting nanovectors in mice bearing EGFR-positive TNBC. Therefore, we envisage that the concomitant administration of cisplatin and siPD-L1 by the nanovectors described in this study may promote synergistic therapeutic effects, along with a reduction in toxic side effects.

## 5. Conclusions

In summary, this study reports the development of a new system for active delivery of siPD-L1 to TNBC cells. In the designed nanosystem, the nanocore was assembled with siPD-L1 and PDL through charge interaction, while the outer shell was conjugated with the sTN145 aptamer for selective targeting of TNBC cells.

The aptamer-conjugated and siRNA-loaded nanoparticles were efficiently taken up by target cells and reached the cytoplasm for suppression of PD-L1 expression.

Our aptamer-based approach provides a safe and efficient tool to target and deliver therapeutic siRNAs to cancer cells with promising potential for targeted applications.

## Figures and Tables

**Table 1:** Properties of polymeric nanoparticles.

PNP Formulation	PNP Characteristics			
	Average Size (nm)	PDI	ζ-Potential (mV)	siPD-L1 Concentration (nM)
empty@PNPs	98.8 ± 0.9	0.179	−32.4	--
empty@PNPs-sTN145	93.9 ± 1.1	0.134	−26.9	--
empty@PNPs-SCR	97.1 ± 1.0	0.182	−21.8	--
siPD-L1@PNPs	101.0 ± 0.2	0.170	−31.8	354.7
siPD-L1@PNPs-sTN145	98.1 ± 0.5	0.159	−23.6	195.3
siPD-L1@PNPs-SCR	98.4 ± 1.9	0.164	−20.2	200.2
FAM-siPD-L1@PNPs	100.7 ± 1.3	0.161	−29.2	334.2
FAM-siPD-L1@PNPs-sTN145	113.1 ± 0.9	0.172	−16.7	200.5
FAM-siPD-L1@PNP-SCR	100.1 ± 0.7	0.166	−20.6	199.8

## Data Availability

Not applicable.

## Supplementary Materials

The following Supporting Information can be downloaded at: https://www.mdpi.com/article/10.3390/pharmaceutics14102225/s1, Figure S1: PD-L1 siRNAs’ testing; Figure S2: Quantitative determination of PD-L1 siRNA entrapped in the nanoparticles; Figure S3: Selective cell uptake and release from endosomes of FAM-siPD-L1@PNPs-sTN145.
