# Supplementary material for: Aptamer-Functionalized Nanoparticles Mediate PD-L1 siRNA Delivery for Effective Gene Silencing in Triple-Negative Breast Cancer Cells
Source: Pharmaceutics. 2022 Oct 18;14(10):2225. doi: 10.3390/pharmaceutics14102225 (PMC9609037; doi:10.3390/pharmaceutics14102225)
Supplement: Supplementary file 1 [file pharmaceutics-14-02225-s001.zip › pharmaceutics-1933694-supplementary.pdf]

Supplementary Materials

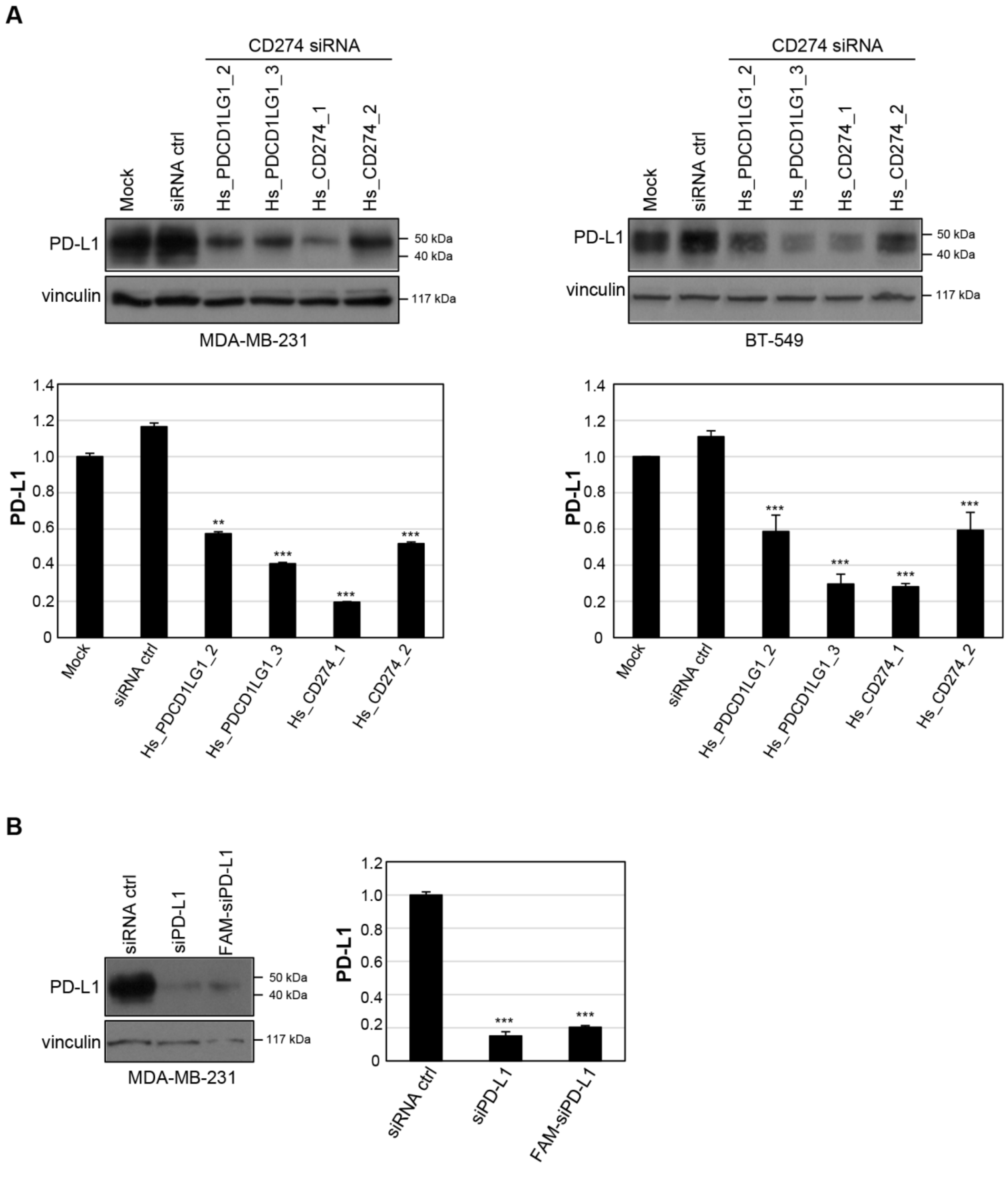

Figure S1. PD-L1 siRNAs testing. MDA-MB-231 or BT-549 cells were mock-treated or transfected with the indicated PD-L1 siRNAs or nonsilencing siRNA (siRNA ctrl) at 30 nM final. Hs_CD274_1 PD-L1 siRNA, naked (siPD-L1) or FAM-labeled (FAM-siPD-L1) were entrapped into PNPs. At 48 h post-transfection, cell lysates were prepared and immunoblotted with anti-PD-L1 antibody, as indicated. Anti-vinculin antibody was used as an immunoblot loading control. Molecular weights of indicated proteins are reported. The histograms indicate PD-L1/vinculin ratio of densitometric signals. Values are shown relative to mock-treated (**A**) or siRNA ctrl (**B**), arbitrarily set to 1. Bars depict means ± SD of three independent experiments. **$P < 0.01$, ***$P < 0.001$.

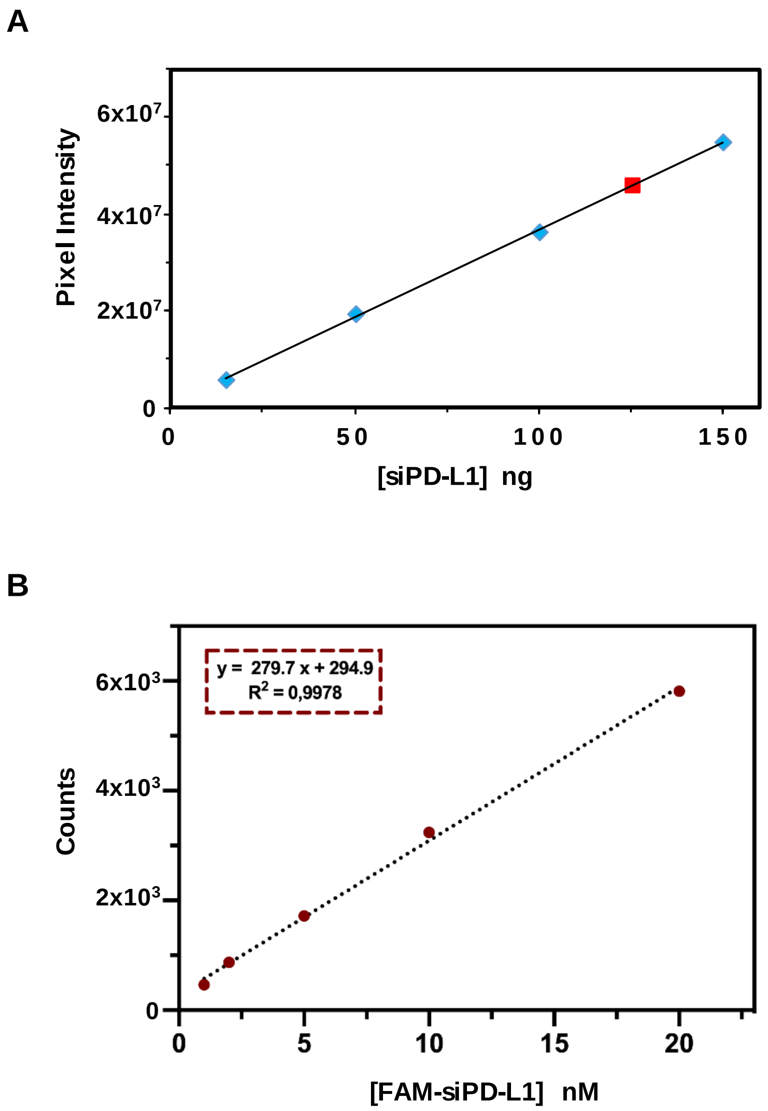

Figure S2. Quantitative determination of PD-L1 siRNA entrapped in the nanoparticles. (**A**) For determination of siPD-L1 extracted from the nanoparticles and loaded onto denaturing PAGE, the calibration line has been obtained by plotting the pixel intensity for a given siRNA band as a function of siPD-L1 amount (ng) loaded onto the gel (blue squares). Red square indicated the amount of extracted siPD-L1 in a typical nanovector formulation. (**B**) Indirect quantification of entrapped siRNA through fluorimetric emission measurement (λ=540 nm) in the wastewater after sample purification. The calibration line has been obtained with the signal measured for different molar concentrations of siRNA, from 1 to 20 nM.

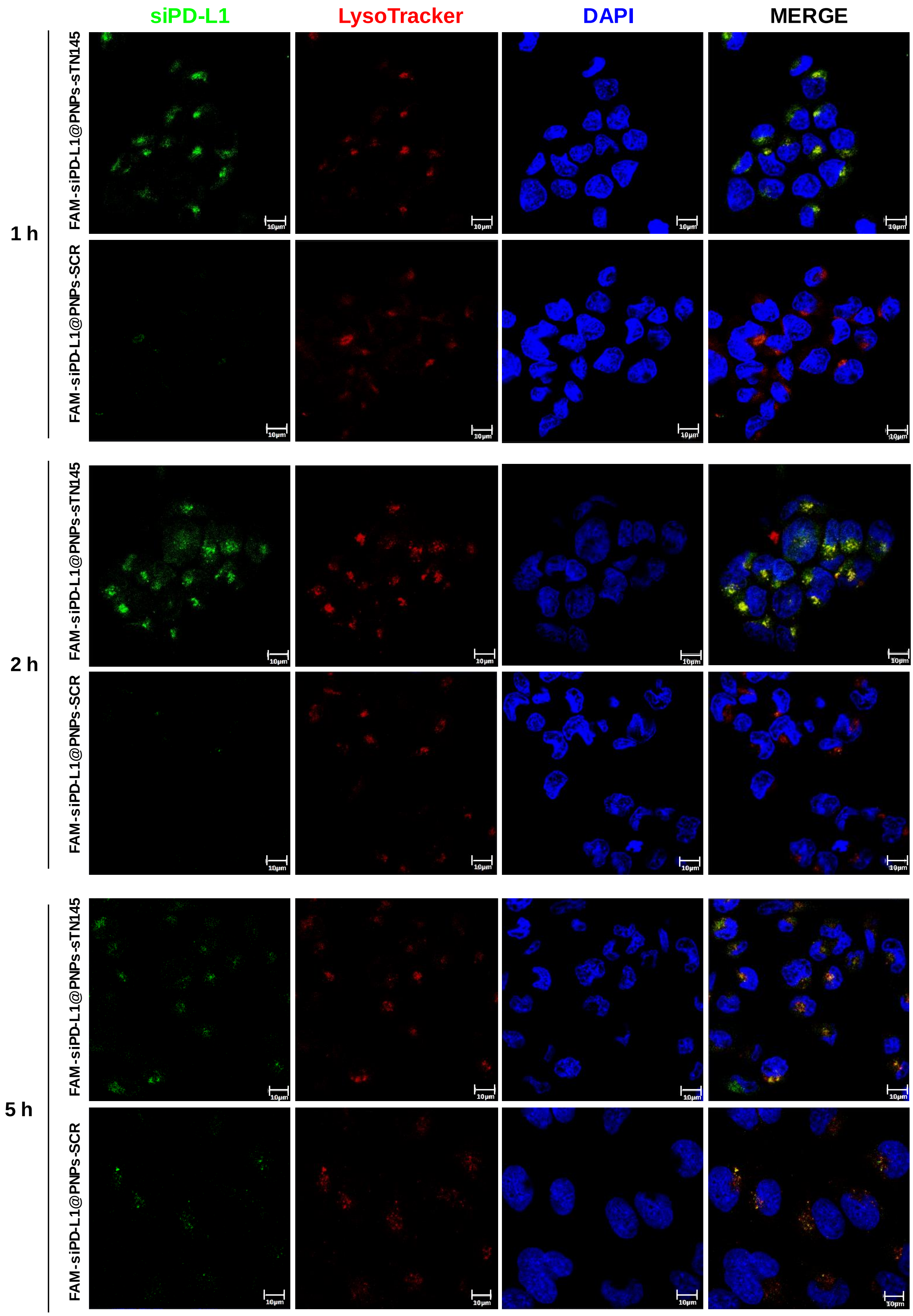

Figure S3. Selective cell uptake and release from endosomes of FAM-siPD-L1@PNPs-sTN145. Single-channel confocal images of representative merged images shown in Figure 4A. FAM-siPD-L1, LysoTracker, and nuclei are visualized in green, red and blue, respectively. Magnification 63×, 1.0× digital zoom, scale bar = 10 μm. All digital images were captured at the same setting to allow direct comparison of staining patterns.
